# Optical sensing of the chemical warfare agent mustard gas and its simulants: a minireview emphasizing fluorescence and colorimetric responses

**DOI:** 10.1039/d6ra07546k

**Published:** 2026-10-08

**Authors:** Dan Meng, Jianan Wei, Liu Yang, Shao Min Shuang

**Affiliations:** a State Key Laboratory of Chemistry for NBC Hazards Protection Beijing 102205 China yangliujinjin@sina.com; b School of Chemistry and Chemical Engineering, Shanxi University Taiyuan 030006 China

## Abstract

Mustard gas, a chemical warfare agent and a type of blister agent, continues to pose a threat to civilian life and public safety. Consequently, there is an urgent need to develop portable, highly sensitive, interference-resistant, and stable detection technologies. Currently, fluorescent probes and colorimetric sensors are emerging as prominent topics in this field, offering advantages such as ease of operation, rapid response times, high sensitivity, and visual detection capabilities. This paper reviews recent advances in fluorescent probes and colorimetric sensors for detecting mustard gas and its simulants, systematically summarizing innovations in reaction types and conditions, recognition sites, response mechanisms, and detection performance. It also addresses current challenges, including the scarcity of gas–solid phase detection methods, the need to improve sensitivity, solvent dependency, and the lack of field detection technologies tailored to real-world scenarios and actual chemical agents. Finally, the paper outlines future research directions.

## Abstract 2

Fluorescent, colorimetric, and their combined methods for the detection of HD and its simulants.

## Graphical abstract

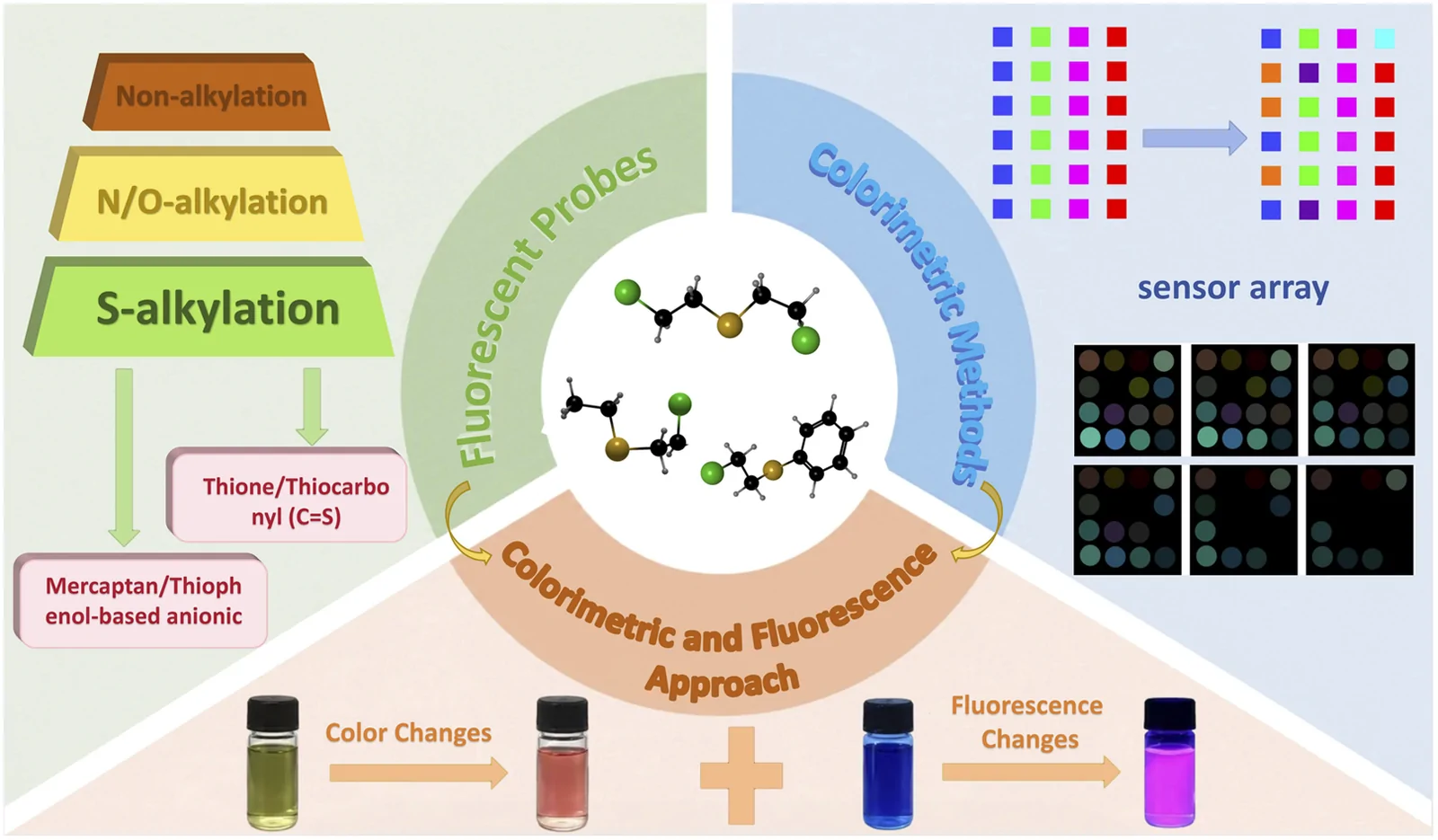

## 1. Introduction

Mustard gas (HD) is a highly toxic chemical warfare agent capable of irreversibly modifying endogenous nucleophiles, including DNA and proteins, through alkylation reactions. The alkylation primarily occurs at the N7 position of DNA purine residues by the chloroethyl group of HD, thereby triggering a series of cytotoxic events that manifest as severe tissue damage to the skin, eyes, and respiratory system. Prolonged or repeated exposure to HD can induce interstrand and intrastrand DNA cross-links, resulting in mutagenic and carcinogenic effects.^1^

Due to its simple synthesis process and the difficulty of preventing its use, coupled with the current lack of a specific antidote or established treatment protocol, HD is frequently employed as a chemical warfare agent in conflicts.^2^ At the same time, the ongoing threat posed by HD and the possibility of its use in terrorist attacks have become focal points of concern for public health and military security.^1^ Consequently, there is a clear and pressing need to develop rapid, reliable detection methods suitable for field deployment.

Traditional detection methods, such as gas chromatography-mass spectrometry (GC-MS), liquid chromatography-mass spectrometry (LC-MS), and ion mobility spectrometry (IMS) can accurately identify mustard gas samples. However, these techniques are generally limited by expensive equipment, complex operations, and cumbersome sample preparation, making it difficult to meet the demands of real-time or on-site detection.^3^ To enhance portability, military and emergency response personnel often use chemical colorimetric test strips, such as M8, M9 and three-way detection strips. However, these colorimetric test strips are susceptible to common interferents—such as acetone, gasoline, and bleach—in complex environments, which can lead to false-positive results.^1^

In contrast, molecular-scale detection of chemical warfare agents can avoid interference from acidic substances and environmental factors, enabling the specific identification of particular toxic gases. Fluorescent probe technology offers high sensitivity, excellent selectivity, rapid response times, and suitability for *in situ* detection, demonstrating significant advantages in chemical warfare agent analysis.^4^ When fluorescent probes interact with chemical warfare agents, molecular structures undergo changes that trigger fluorescence enhancement or quenching, with detection limits reaching the micromolar or even nanomolar range. Meanwhile, colorimetric sensors, which do not rely on complex instrumentation and allow for direct interpretation of results through color changes, demonstrate excellent practical value for rapid screening at emergency sites.^5^ Both fluorescent and colorimetric sensors share advantages such as controllable costs, ease of operation, and suitability for miniaturization, providing a promising technical pathway for field detection of mustard gas and its simulants.

It is worth noting that in recent years, several review articles have addressed research in the field of mustard gas sensing. Li *et al.* systematically reviewed progress in fluorescent probes for mustard gas detection, classifying them based on detection strategies.^6^ Kumar *et al.* provided a comprehensive review from the broader perspective of chemical warfare agent detection.^7^ Compared with these studies, the distinctive contributions of this review are primarily reflected in three aspects. First, in terms of organizational logic, this paper classifies content based on the reaction mechanisms of *S*-alkylation, *N*/*O*-alkylation, and noncovalent interactions, rather than listing studies chronologically or by detection strategy. This mechanism-driven framework helps readers understand the chemical principles underlying different probe designs—for example, how thiophenol probes can react under neutral conditions due to their low p*K*_a_, how aliphatic thiols require strong base activation, and how ketone probes circumvent the inherent susceptibility of thiols to oxidation. Second, regarding content coverage, while previous reviews have focused on fluorescence sensing, this paper symmetrically covers both colorimetric methods and “fluorescence + colorimetric” dual-mode detection.

This review examines methods such as the classic NBP method, the thiosulfate/iodine/starch system, and AuNPs colorimetric arrays, thereby addressing a gap in existing literature. More importantly, it is the first paper to systematically highlight that over 90% of current studies rely solely on CEES for validation. However, as a monofunctional alkylating agent, CEES cannot form the ternary cyclopentadienyl-pyridinium intermediate characteristic of true HD. There are fundamental differences in the reaction kinetics between the two. Consequently, many ultra-sensitive probes optimized for CEES often exhibit reduced detection performance when applied to true HD, which is less reactive. From an application-oriented perspective, this paper focuses on the challenges sensors face in transitioning from laboratory research to field deployment, including issues such as long-term storage stability and reusability—topics that have not yet been systematically addressed in existing reviews. In summary, this review aims to provide a new analytical framework based on three dimensions—reaction mechanisms, the suitability of simulants, and engineering bottlenecks—with the goal of advancing the practical transformation of mustard gas optical sensing technology from laboratory research to field detection equipment.

## 2. Types and differences among mustard gas simulants

HD is a bifunctional alkylating agent whose toxicity primarily arises from the nablyl effect mediated by the two chlorine atoms at either end of the molecule. On moist surfaces or in body fluids, the departure of one chlorine atom triggers the sulfur atom within the molecule to immediately attack the carbon at the opposite end, forming a highly reactive three-membered ring sulfonium ion intermediate. This intermediate then reacts with two guanine bases on the DNA double helix, forming intrastrand or interstrand crosslinks that prevent DNA strands from separating during replication, ultimately leading to cell death, carcinogenesis, and genetic damage. Due to its extreme toxicity and regulatory restrictions, numerous studies have employed structurally similar analogs as substitutes for experimental purposes.^3^ These analogs can be classified into three categories based on their structural characteristics and differ from HD in key ways.

First, there are the monochlorothioether compounds, including 2-chloroethyl ethyl thioether (CEES), 2-chloroethyl methyl thioether (CEMS) and 2-chloroethyl phenyl thioether (CEPS). These compounds retain only one chlorine atom, with an alkyl or aryl group at the other end. Mechanistically, they act as monofunctional alkylating agents. After the chlorine atom departs, they form open sulfur or carbon cation intermediates. Unlike HD, they cannot form three-membered ring structures. Therefore, they can only attack a single site on DNA at a time, resulting in the alkylation of a single base. This makes them easily repairable by cells and prevents the formation of lethal inter-strand crosslinks. In terms of toxicity, these monochlorinated analogs still cause skin blistering and exhibit cytotoxicity, classifying them as moderately toxic compounds. The methyl group in CEMS is too small, making them highly volatile (low boiling point) which makes concentration control difficult in experiments and leads to equipment contamination. CEPS's phenyl group is too sterically hindered. Its large size impedes the departure of the chlorine atom and subsequent reactions, resulting in a reaction rate that is much slower than that of CEES. This can lead to “false negatives” in experimental results or an underestimation of toxicity. CEES possesses a complete structure of both the sulfide and chlorine atoms. This allows it to undergo key alkylation reactions and simulate most of the cytotoxic mechanisms induced by HD such as inflammation, oxidative stress, and DNA single-strand breaks. Therefore, it is widely used in laboratory research.

Next are the non-chlorinated thioethers, such as diethyl sulfide (DES), ethyl methyl sulfide (EMS) and thiodiglycol (TDG). These compounds completely lack the leaving groups necessary for alkylation and do not form covalent bonds with nucleophiles under physiological conditions. Among them, TDG is the primary hydrolysis product of HD and is commonly used as a marker in environmental monitoring. These simulants exhibit extremely low toxicity; DES is even used as a food additive.

Finally, there are non-sulfide ester simulants such as methyl salicylate (MS), ethyl benzoate (EB), and diethyl adipate (DEA). These analogs contain neither sulfur nor chlorine atoms and are incapable of undergoing alkylation reactions. Their only connection to HD lies in the similarity of physical properties such as volatility and refractive index. Therefore, they are suitable only for applications unrelated to chemical reactions, such as chromatographic column calibration and testing of sensor physical responses. Their toxicity is negligible.

In summary, the extreme toxicity of HD arises from its dual-alkylating capability, which is conferred by its dichlorinated structure. While monochlorinated analogs can partially replicate its reactivity, their toxicity is significantly reduced. Chlorine-free and non-sulfur analogs do not undergo alkylation reactions and are used solely as physical references. These differences determine the scope of application for various simulants in research, indicating that analogs such as monochlorosulfides can be employed to investigate toxicological mechanisms and protective materials, whereas ester compounds are suitable only for non-reactive tests, such as instrument calibration.

See Table 1 for details on the differences between mustard gas and its simulant.

**Table 1:** Structures and characteristics of mustard gas and its simulants

Compound	Abbreviation	Structural formula	Structural features	Alkylation type	Toxicity level
Bis(2-chloroethyl)sulfide	HD	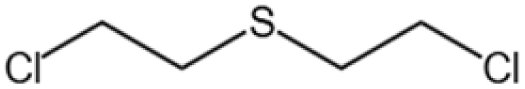	Dichlor	Dual-function alkylating agent	Highly toxic
			Cl–CH_2_CH_2_–S–CH_2_CH_2_–Cl		
2-Chloroethyl ethyl sulfide	CEES	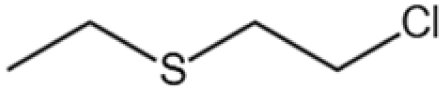	Monochloro + ethyl	Monofunctional alkylating agent	Moderately toxic
			Cl–CH_2_CH_2_–S–CH_2_CH_3_		
2-Chloroethyl methyl sulfide	CEMS	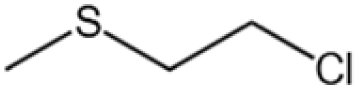	Monochloro + methyl	Monofunctional alkylating agent	Moderately toxic
			Cl–CH_2_CH_2_–S–CH_3_		
2-Chloroethyl phenyl sulfide	CEPS	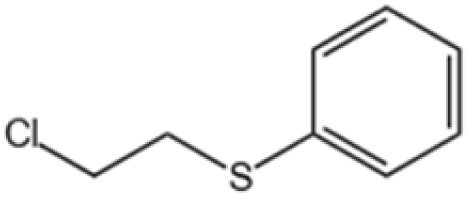	Monochloro + phenyl	Monofunctional alkylating agent	Moderately to slightly toxic
			Cl–CH_2_CH_2_–S–Ph		
Thiodiglycol	TDG	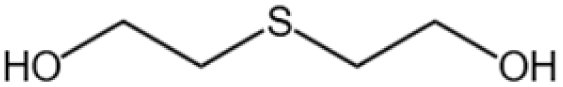	Dihydroxy	No alkylation occurs	Low toxicity
			HO–CH_2_CH_2_–S–CH_2_CH_2_–OH		
Diethyl sulfide	DES	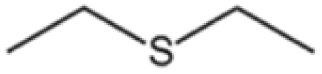	Chlorine-free + diethyl	No alkylation occurs	Low toxicity
			CH_3_CH_2_–S–CH_2_CH_3_		
Ethyl methyl sulfide	EMS	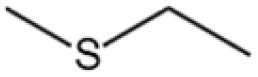	Chlorine-free + ethyl + methyl	No alkylation occurs	Toxic-free
			CH_3_CH_2_–S–CH_3_		
Methyl salicylate	MS	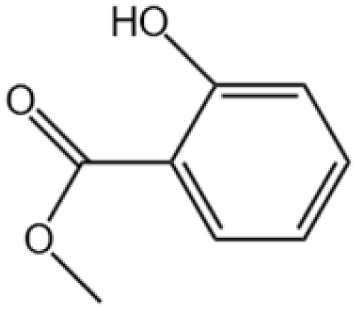	Sulfur-free and chlorine-free	No alkylation occurs	Toxic-free
			Esters containing benzene rings		
Ethyl benzoate	EB	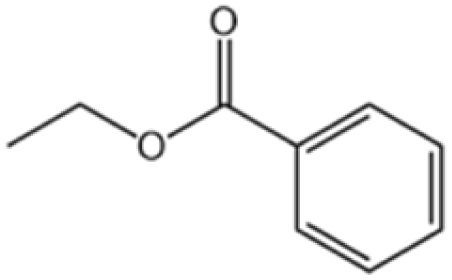	Sulfate-free and chlorine-free	No alkylation occurs	Low toxicity
			Aromatic diester		
Diethyl adipate	DEA	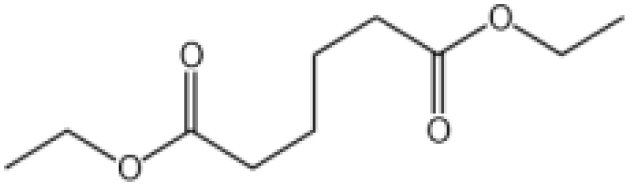	Sulfate-free and chlorine-free	No alkylation occurs	Toxic-free
			Aliphatic diester		

## 3. Key performance parameters

A series of key performance parameters serve as fundamental criteria for evaluating practical application potential of optical sensors. Sensitivity, typically characterized by the limit of detection (LOD), determines the lowest target concentration that the sensor can detect and is the primary indicator for addressing extremely low exposure risks associated with HD. Selectivity (or resistance to interference) ensures that the sensor produces a specific response to the target substance in complex environments, such as those containing other volatile organic compounds, nerve agent analogs or high humidity, thereby preventing false alarms. Response time is critical for the timeliness of early warnings; an ideal sensor should generate a signal output within minutes or even seconds. In addition, a sensor's adaptability to different detection media—its ability to function effectively in gaseous, liquid, solid (such as test strips), or film forms—is directly linked to its deployment scenarios. Different technical solutions are required for tasks ranging from the analyzing of hydrolysis products of toxic agents in solutions to real-time monitoring of vapor phases in the air.

## 4. A method for detecting mustard gas and its simulants using “open-type” fluorescent probes

In polar media, HD can spontaneously form a three-membered ring thioanion through the involvement of the group adjacent to the sulfur atom, as shown in Fig. 1. In this structure, the methylene group functions as an electrophilic site and exhibits strong reactivity toward certain nucleophiles. HD possesses strong alkylating capabilities and reacts readily with thiols or tertiary amines. Leveraging this property, many probes containing thiols or thiones have been developed based on HD-induced nucleophilic reactions, which generate distinct optical signals^8^ and offer advantages such as high signal-to-noise ratios and enhanced sensitivity.

**Fig. 1:**
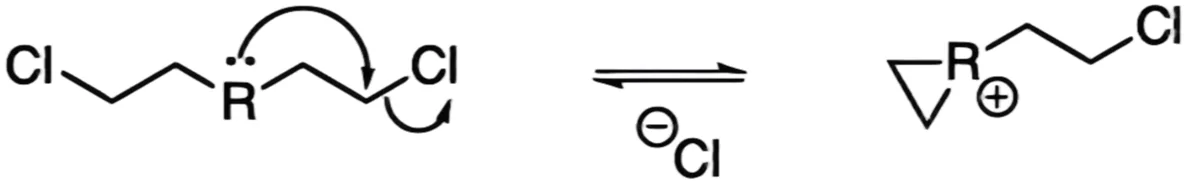
Mustard gas and nitrogen mustard form three-membered ring cations *via* intramolecular nucleophilic attack.

### 4.1 Fluorescent probes designed based on *S*-alkylation reactions

#### 4.1.1 Thiol/thiophenolate-based anionic probes

Kumar and Anslyn reported the first thiol-based fluorescent probe. They designed a fluorescent sensor for a multicomponent detection system that utilizes the nucleophilicity of thiols to detect CEES in aqueous solutions (see Fig. 2(a)).^9^ In the detection reaction, the disulfide compound 1 reacts with CEES to form the tetrasulfide podand 2. Taking advantage of podand 2's higher affinity for Cd^2+^, it displaces 4-methylcoumarin (ME) from the non-fluorescent complex 8 formed between Cd^2+^ and the fluorescent indicator ME, thereby restoring fluorescence. This enables a rapid response to CEES within 1 minute at 80 °C, with a detection limit of 0.2 mM. To improve detection selectivity, they used 4-phenyl-3-butyn-2-one to “cap” unreacted compound 1, thereby preventing false-positive signals caused by compound 1 binding to Cd^2+^ and displacing ME to generate fluorescence. At the same time, the method demonstrated good selectivity toward interferents such as 2-chloroethyl ethyl ether, acetyl chloride, and the nerve agent analog DEP, and was successfully applied to the detection of CEES in surface swab samples and spiked soil samples. This work represents the first thiol-based fluorescent detection method for CEES and marks the first application of the metal ion indicator displacement (IDA) strategy to CEES detection, providing important proof of concept and a theoretical basis for the subsequent design of probes based on thioether coordination chemistry.

**Fig. 2:**
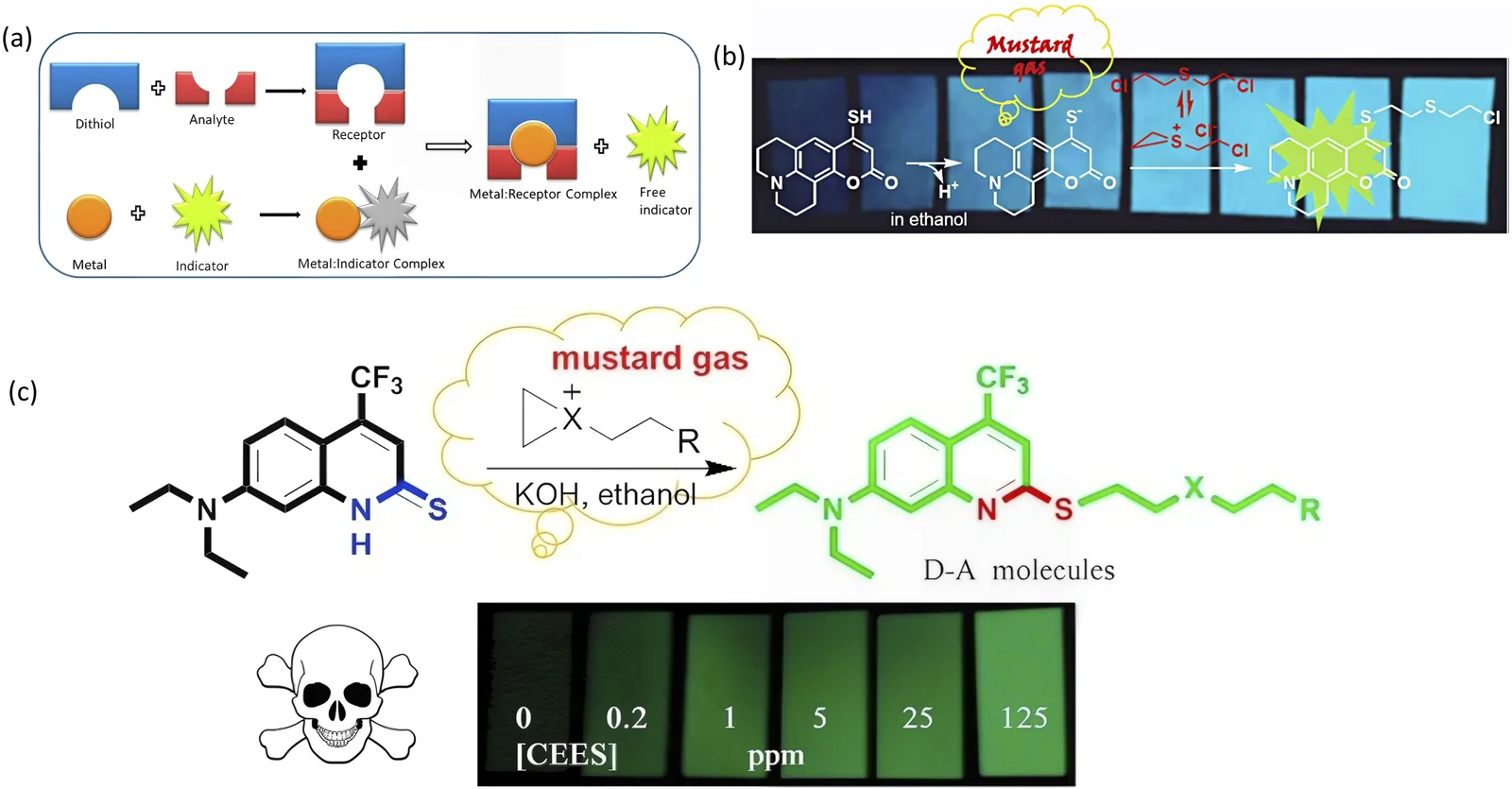
(a) Kumar and Anslyn reported the first thiol-based fluorescent probe; (b) Et-Cou-SH and Pr-Cou-SH designed by Wei *et al*.; (c) Feng *et al.* developed fluorescent probes (2a–2d).

To simplify the detection system and enhance sensitivity, Wang *et al.* directly linked thiol groups to a tetraphenylethylene (TPE) backbone possessing aggregation-induced emission (AIE) properties, achieving signal amplification through Hg^2+^-mediated “sandwich” stacking-induced fluorescence.^10^ The detection principle involves constructing probe S4 by modifying the TPE backbone with four thiol-substituted phenyl groups. S4 itself exhibits almost no fluorescence in dilute solution. After the four benzenethiol groups on S4 undergo nucleophilic substitution reactions with CEES, a four-armed, open-chain product, L, is formed. In the presence of Hg^2+^, this product forms a “sandwich”-type 2 : 1 complex, which restricts the rotation of the TPE benzene rings, thereby activating the AIE effect and significantly enhancing fluorescence. The detection limit is 1.10 µM. Additionally, due to a reversible phase transition between the crystalline and amorphous states, S4 emits blue fluorescence in the crystalline state and green fluorescence in the amorphous state; and this transition can be reversibly restored by heating (97 °C). This study marks the first application of the AIE concept to HD detection, achieving approximately a 22-fold fluorescence enhancement.

As shown in Fig. 2(c), Feng *et al.* developed fluorescent probes (2a–2d) based on a quinoline-2-thione skeleton. In the presence of KOH, thione/thio-mercaptan tautomerism occurs, generating a highly nucleophilic thiol anion *in situ*.^11^ Following *S*-alkylation with HD, this forms a D–A-structured thioether product, resulting in significantly enhanced fluorescence. Probes 2b and 2d, featuring 4-trifluoromethyl and 6/7-diethylamino groups respectively exhibited, optimal performance, responding to CEES and nitrogen mustard (NH1) in less than 1 minute at 60 °C, with detection limits of 90 nM and 20 nM, respectively. Meanwhile, their response time to HD was approximately 4.5–5 minutes, with a detection limit of 50 nM, outperforming most contemporaneous reports. Additionally, the study developed polyethylene oxide film test strips capable of detecting gaseous CEES at concentrations as low as 0.2 ppm within 4 minutes, providing a portable tool for on-site detection. This work elucidates the effects of substituents on the p*K*_a_, response speed, and fluorescence properties of the probes, offering important guidance for the rational design of quinolinesulfonate probes.

To avoid the use of external bases and enhance detection sensitivity and reaction rates, Wei *et al.* leveraged the low p*K*_a_ of thiophenol to design two 4-mercapto-coumarin-based fluorescent probes (Et-Cou-SH and Pr-Cou-SH), as depicted in Fig. 2(b).^12^ Spontaneous deprotonation in ethanol generates a thiophenol anion, enabling it to act as a strong nucleophile that undergoes *S*-alkylation reactions with HD without the need for added alkali, thereby restoring coumarin fluorescence after the formation of thioether products. Among these, Pr-Cou-SH demonstrated best performance, with response times of 1.5, 1 and 4 minutes for CEES, NH1 and SM respectively at 60 °C and detection limits of 6.6 nM, 3.6 nM, and 16 nM, respectively. Importantly, the triethylamine (TEA) added in the study preferentially reacts with reactive electrophiles such as DCP, acyl chloride, and POCl_3_, preventing their nonspecific reactions with the probe. For gas-phase detection, the research team prepared polyethylene oxide film test strips containing trioctylamine (TOA) capable of detecting CEES vapor at concentrations as low as 2.5 ppm within 3 minutes, demonstrating excellent storage stability and selectivity (Table 2).

**Table 2:** Probes based on thiol/thiophenol anions

Author	Probe name	Reaction type	Reaction solvent	Temperature	LOD	Response time	Fluorescence	Real chemical agents
Kumar and Anslyn^9^	Dithiol 1 + Cd^2+^/ME	Thiol alkylation	Water (pH = 9)	80 °C	0.2 mM (CEES)	1 min	Blue	—
Wang *et al.*^10^	S4	Thiol alkylation	DMSO/buffer solution pH = 9	80 °C	1.10 µM	5 min	Green	—
Feng *et al.*^11^	2a–2d	Thiophenolate alkylation	Ethanol/gas phase (test strip)	60 °C/RT (test strip)	CEES: 90/SM: 50/NH1: 20 nM/0.2 ppm (test strip)	CEES/NH1: <1 min/SM: 4.5–5 min/4 min (test strip)	Green/blue	HD
Wei *et al.*^12^	Et-Cou-SH, Pr-Cou-SH	Thiophenolate alkylation	Ethanol/gas phase (test strip)	60 °C/RT (test strip)	CEES: 6.6/SM: 16 nM/NH1: 3.6/9 ppb (test strip)/naked eye: 2.5 ppm (test strip)	CEES: 1.5 min/SM: 4 min/NH1: 1 min/3 min (test strip)	Cyan	HD

#### 4.1.2 Thioketone/thionyl (C=S) probes

In the detection of mustard gas and its analogs, although thiol- and thiophenol-based probes exhibit high reactivity, they almost universally require strong bases and heating. Moreover, thiols are easily oxidized by oxygen under ambient conditions, forming less reactive disulfides.^4^ In contrast, ketone-based probes feature a more sophisticated design and represent a superior molecular engineering strategy (Table 3). Some can operate at room temperature without the need for bases, making them more suitable for field applications. In thiocarbonyl-based probes, the thiocarbonyl group serves as the reactive moiety, thereby avoiding the undesirable oxidation of thiols.

**Table 3:** Probes based on thioketones and thioketal derivatives

Author	Probe name	Reaction type	Reaction solvent	Temperature	LOD	Response time	Fluorescence	Real chemical agents
Zhang *et al.*^13^	DPXT	Thiocarbonyl alkylation	DCM/gas phase (TLC silica gel plate)	RT	1.2 µM/0.5 ppm (TLC silica gel plate)	1 min (TLC silica gel plate)	Pink	—
Meng *et al.*^14^	SM-Flu	Thioamide alkylation	PBS buffer (10% MeCN)	37 °C	1.20 µM	∼30 min	Green	HD
Meng *et al.*^15^	OxSM-1,2,3, SiNIR-SM	Thioketone alkylation	PBS buffer (20% CH_3_CN)	37 °C	SM: 41 nM	SM: 1.5 min (60 °C)	Near-infrared/red	HD
Ma *et al.*^4^	DPXT	Thiocarbonyl alkylation	Water (polymer suspension)	RT	0.9 µM (SM)	SM: 10 min	Pink	HD
Feng *et al.*^16^	AQso	*Ortho*-hydroxythione bisite	Acetonitrile/ethanol	60 °C	SM: 70 nM	SM: 40 min (RT)	Cyan/blue	HD
Yan *et al.*^17^	Cy3C=S	Thiocarbonyl alkylation	Methanol/isopropyl ether (9 : 1, v/v)	60 °C	0.44 µM	40 min	Purple/blue	—

In the early stages of development, Zhang *et al.* pioneered the synthesis of the probe DPXT (3,6-di(piperidin-1-yl)-9*H*-xanthene-9-thione) using 3,6-dihydroxyxanthone as the starting material (Fig. 3(b)). They introduced two electron-donating piperidine groups at the 3- and 6-positions and replaced the carbonyl group at the 9-position with a thiocarbonyl group (C=S) using Lawesson's reagent, yielding a thiopyronin-type dye.^13^ Utilizing the thiocarbonyl group as a nucleophilic site, a nucleophilic reaction with CEES produces a thio-pyridinium derivative, resulting in a fluorescence enhancement factor as high as 850-fold. The detection limit for CEES in solution is 1.2 µM, and the probe can detect CEES vapor at concentrations as low as 0.5 ppm within 1 minute in the gas phase. Furthermore, the probe was successfully applied to a TLC plate-based vapor detection platform, demonstrating excellent recognition specificity.

**Fig. 3:**
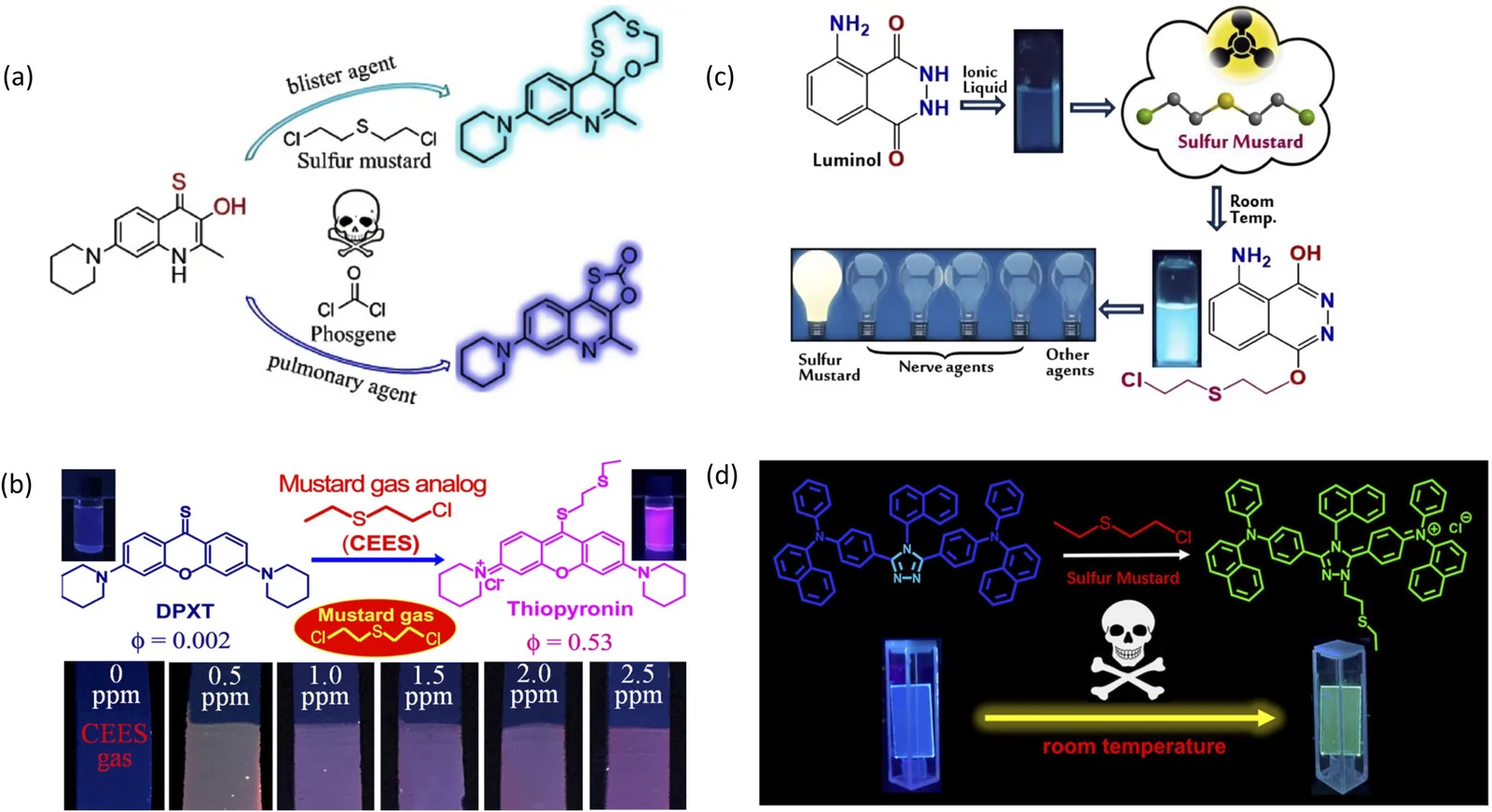
(a) Feng *et al.* designed the AQso probe; (b) the DPXT probe (3,6-di(piperidin-1-yl)-9*H*-xanthene-9-thione) designed by Zhang *et al.*; (c) the fluorescence sensor based on luminol (LH_2_) and the ionic liquid [emim][DCA] fabricated by Singh *et al.*; (d) Zheng *et al.* designed NDTAZ.

Meng *et al.* developed a fluorescent probe named SM-Flu, using fluorescein as the fluorophore and introducing a thioamide as the recognition group. This became the first probe capable of real-time detection of HD in live cells and *in vivo*.^14^ Furthermore, the study reported, for the first time, the concentration–time distribution of HD in mouse brain tissue and found that HD rapidly enters the brain and persists for up to 48 hours. In the same year, they developed a series of fluorescent probes for early HD diagnostic imaging based on thioketone chemistry, significantly improving the detection limit and response time.^15^ Among these, SiNIR-SM was the first near-infrared fluorescent probe to demonstrate the fastest response speed and highest signal-to-noise ratio. It enabled the screening of scavengers such as NaHS and glutathione, which effectively reduce intracellular HD concentrations, and was the first to reveal that HD may accumulate in mitochondria. More importantly, SiNIR-SM can still detect specific fluorescent signals within 2 hours after SKH-1 mouse skin is exposed to HD, thus offering unique advantages for the early diagnosis of HD poisoning.

However, these probes still rely on organic solvents, which makes them difficult to apply directly to surface contamination detection in real-world environments. To address this limitation, Ma *et al.* developed a sprayable method for visualizing HD surface contamination by loading the known probe DPXT onto a hydrophobic polyacrylonitrile polymer and dispersing it in an aqueous polyvinylpyrrolidone solution.^4^ After establishing an HD contamination model on a painted metal surface, they sprayed the material and, under illumination with a 365 nm UV lamp, were able to clearly distinguish the boundary between contaminated and safe areas with the naked eye within 10 minutes, with fluorescence enhanced by approximately 22-fold. This study provides a practical solution for *in situ*, non-destructive, and visual detection of HD surface contamination, offering promising applications in battlefield environment assessment and contamination screening of sensitive equipment.

The work by Feng *et al.* represents a significant advancement in thioketone probes, progressing from “single-target detection” to “multi-target discriminatory detection.” As presented in Fig. 3(a), by using 7-aminoquinoline as the fluorophore and an *o*-hydroxythioketone as the dual reaction site, they developed AQso, the first bifunctional fluorescent probe capable of simultaneously detecting and distinguishing between HD and phosgene.^16^ The sulfur atom initially undergoes *S*-alkylation with HD, followed by *O*-alkylation of the oxygen atom, yielding a cyclic ether/thioether product. This reaction results in fluorescence enhancement and cyan light emission, with a detection limit of 70 nM. The *ortho*-hydroxythione undergoes an acylation reaction with phosgene to form a cyclic carbonate structure, leading to fluorescence enhancement and blue emission with a detection limit of 14 nM. At the same excitation wavelength (370 nm), HD and phosgene induce fluorescence at different emission wavelengths, enabling selective differentiation and detection. Regarding selectivity, AQso demonstrates a significantly superior response to HD and phosgene compared to other alkyl halides, acyl halides, nerve agent analogs and common interferents. Furthermore, portable test strips prepared by loading AQso onto melt-blown nonwoven fabric, combined with a smartphone RGB color application, enable semi-quantitative detection of gaseous CEES and phosgene within a range of 0–50 ppm. As the first dual-functional fluorescent probe capable of distinguishing between HD and phosgene, this AQso offers a novel strategy for multi-target detection in complex chemical warfare agent environments.

Building upon the aforementioned thione-based probes built on conventional fluorophores, Yan *et al.* for the first time introduced cyanine dyes into the fluorescent detection of CEES and developed the probe Cy3C=S.^17^ Its synthesis begins with the condensation of 2-methylchromone and Fischer's aldehyde to construct an asymmetric trimethine cyanine scaffold (Cy3C=O), which is subsequently thionated with Lawesson's reagent to afford the target probe Cy3C=S. The thiocarbonyl group serves as the nucleophilic reactive site; upon *S*-alkylation with CEES, the probe undergoes ring-opening to generate Cy3CEES, accompanied by a distinct color change from purple to blue and a 106-fold fluorescence enhancement at 655 nm. The assay proceeds in methanol/isopropyl ether (9 : 1) at 60 °C, reaching completion within 40 min, with a detection limit of 0.44 µM and excellent linearity over 0–100 µM (*R*^2^ = 0.9995). Selectivity tests reveal negligible fluorescence responses toward nerve agent mimics (DCP, DCNP), oxygen mustard analogs (BCEE, CEEE), and halogenated hydrocarbons (IE, BCP). Notably, the reaction proceeds without the addition of base or catalyst, simplifying the operational procedure. The probe has also been validated in CEES-contaminated soil samples, where a fluorescence enhancement is observable within 2 min of heating after methanol extraction, affording a detection limit of ∼8 µM in soil matrix, thereby demonstrating its practical utility in real environmental samples. As the first example of a cyanine-based probe for sulfur mustard simulant detection, this work expands the palette of available fluorophores and establishes asymmetric cyanine dyes as a viable platform for chemical warfare agent sensing.

### 4.2 Fluorescent probes designed based on other alkylation (*N*/*O*-alkylation) reactions

In addition to the *S*-alkylation-based fluorescence detection methods described above, researchers have developed fluorescence detection techniques that target *N*- or *O*-nucleophilic sites (Table 4). These methods exploit alkylation reactions between mustard gas (and its simulanus) with nitrogen- or oxygen-containing functional groups, inducing changes in the fluorescence signal. This approach enables effective identification and quantitative analysis of target molecules.

**Table 4:** Probes based on other alkylation reactions

Author	Probe name	Reaction type	Reaction solvent	Temperature	LOD	Response time	Fluorescence	Real chemical agents
Singh *et al.*^18^	Luminol + ionic liquid [emim][DCA]	*O*-Alkylation	Bicarbonate-hydroxide buffer	RT	6 ppm (SM)	<60 s	Light blue → sky blue → green (naked eye)	HD
Zheng *et al.*^19^	Triazole-based AIEE material NDTAZ	*N*-Alkylation	Gas phase	RT	0.55 ppm	<1 s	Blue → green	—

Fig. 3(c) shows a fluorescence sensor based on luminol (LH_2_) and the ionic liquid [emim][DCA] first reported by Singh *et al.*^18^ Under basic conditions, LH_2_ forms a monosodium salt and its enolic structure exhibits enhanced nucleophilicity due to the presence of [emim][DCA]. The chloroethyl group of HD undergoes an *O*-alkylation reaction with the oxygen atom of LH_2_, yielding a conjugation-enhanced *O*-alkylated product. This reaction results in a significant increase in fluorescence with a detection limit of 6 ppm and a response time of less than 1 minute.

Inspired by the critical influence of electron density at the nucleophilic site on reaction rates, Zheng *et al.* adopted a novel approach by shifting the oxygen nucleophilic site from oxygen to nitrogen. As demonstrated in Fig. 3(d), they designed an aggregation-induced emission enhancement (AIEE) material, NDTAZ, based on a triazole (TAZ) unit.^19^ In this material, the 1,2,4-triazole unit serves as the nucleophilic active center, with strong electron-donating *N*-phenyl-1-naphthylamine (NPN) groups attached on both sides and a naphthalene ring introduced at the 4-position to enhance solid-state luminescence efficiency. The nitrogen atom in NDTAZ undergoes nucleophilic substitution with CEES, yielding a stable addition product. This reaction disrupts the original intramolecular charge transfer (ICT) state and forms a new ICT state, resulting in a response time of less than 1 second and a detection limit as low as 0.55 ppm. Subsequent water treatment hydrolyzes the C–N bond, releasing CEES and restoring the original fluorescence. Furthermore, the material has been formulated into fluorescent films and filter paper test strips for ultrafast, highly selective detection of CEES vapor at room temperature.

### 4.3 Fluorescent probes designed based on non-covalent interactions/physical adsorption

As shown in Fig. 4(a), Qiu *et al.* designed self-assembled and co-assembled microsphere sensor arrays using two types of fluorene-based oligomers through intermolecular noncovalent interactions (sulfur–π and dipole–dipole), successfully achieving highly sensitive and selective detection of HD. This work demonstrates the immense potential of supramolecular chemistry in mustard gas sensing.^20^ The study found that self-assembled microspheres composed of a single oligomer 1 exhibited full-spectrum fluorescence enhancement toward HD, while displaying orthogonal fluorescence responses toward other sulfides. In contrast, co-assembled microspheres formed by oligomer 1 and oligomer 2 (containing benzothiadiazole units) exhibited rapid fluorescence quenching in response to HD. Through the differentiated response patterns of the two materials, selective detection of HD was successfully achieved with a detection limit as low as 30 ppb and a response time of approximately 4 seconds. This work represents the first application of noncovalent interactions for the fluorescent detection of HD, avoiding the long reaction times required by traditional nucleophilic substitution reactions and offering advantages such as rapid response, high sensitivity, and the capability to be arrayed for recognition.

**Fig. 4:**
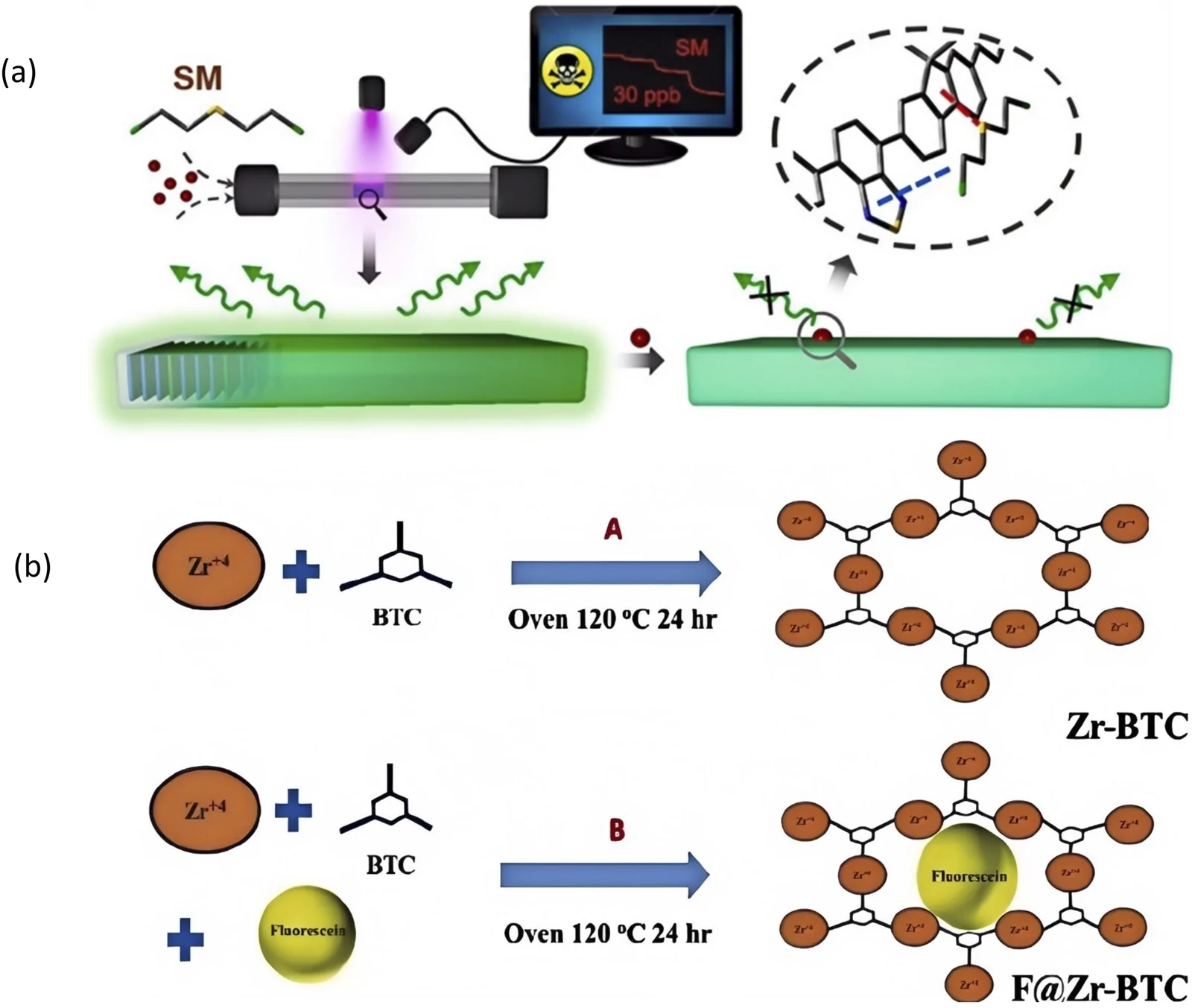
(a) Qiu *et al.* designed self-assembled and co-assembled microsphere sensor arrays; (b) Abuzalat *et al.* encapsulated fluorophores *in situ* within the pores of Zr-BTC.

However, in practical applications, purely organic materials often encounter challenges such as insufficient photostability and limited active site density. In response, Abuzalat *et al.* focused on metal–organic frameworks (MOFs), which possess high specific surface areas and well-ordered pore channels. Using a simple and rapid solvothermal method, they successfully encapsulated fluorophores *in situ* within the pores of a zirconium-based MOF (Zr-BTC), thereby creating a novel fluorescent probe F@Zr-BTC, as illustrated in Fig. 4(b).^21^ The material emits strong fluorescence at 534 nm when excited at 470 nm. Upon exposure to CEES, the electron-rich CEES molecules undergo electron transfer with the electron-deficient fluorophore in F@Zr-BTC, resulting in fluorescence quenching. The detection limit is as low as 48 ppb, and the probe demonstrates good selectivity toward CEES, H_2_S, 2-mercaptoethanol, and DMMP. The advantages of this method include rapid synthesis, simple operation, and high sensitivity, offering new insights into the application of MOF-based fluorescent sensors for detecting of simulated chemical warfare agents (Table 5).

**Table 5:** Probes based on non-covalent/physical adsorption

Author	Probe name	Reaction type	Reaction solvent	Temperature	LOD	Response time	Fluorescence	Real chemical agents
Qiu *et al.*^20^	Fluorenyl oligomer 1 and its co-assembled microspheres with 2	Non-covalent interactions	Gas phase	RT	CEES: 10 ppb/SM: 30 ppb	SM: ∼4 s	Blue → green	HD
						CEES: ∼5 s		
Abuzalat *et al.*^21^	F@Zr-BTC	Electron transfer	Ethanol	RT	48 ppb	—	Yellow-green → quenching	—

## 5. Detection of mustard gas and its simulants using colorimetric methods

The key advantages of the colorimetric method for detecting mustard gas and its simulants are in its visual, rapid, simple, and cost-effective nature, making it particularly suitable for rapid on-site screening (Table 6).

**Table 6:** Detection of mustard gas and its simulants using a colorimetric method

Author	Color-rendering system	Reaction type	Reaction solvent	Temperature	LOD	Response time	Fluorescence	Real chemical agents
Kangas *et al.*^22^	Colorimetric sensor array	Multiple non-covalent and covalent interactions	Mixed solvent of acetic acid buffer/ethylene glycol/triethylene glycol monobutyl ether/glycerol	RT	—	—	—	—
Zhou *et al.*^1^	NBP and its derivatives	Nucleophilic substitution	Acetone/water mixed solvent	RT/60 °C	Naked eye: 10 ppm (RT)/2.5 ppm (60 °C)	<10 s (RT/add alkali)	Colorless/pale yellow → blue/purple	HD
Yadav *et al.*^23^	Sodium thiosulfate–iodine-starch system	Nucleophilic substitution	Water/ethanol mixture	RT	Naked eye: 0.02 mg	5 min	Colorless → dark blue	HD, NM
					Spectroscopic analysis: 0.002 mg			
Xu *et al.*^5^	Gold nanoparticles (AuNPs) of four different particle sizes	Au–S coordination-induced aggregation	Aqueous phase	45 °C	4.13 mg·L^−1^	6 min (45 °C)	Red → purple/pink/sky blue	HD

Kangas *et al.* developed a detection method based on a colorimetric sensor array to identify seven chemical warfare agent simulants, including simulants of HD and nerve agents.^22^ Of the 188 colorimetric sensors screened, they successfully distinguished between different simulants by extracting RGB values and applying principal component analysis (PCA) to reduce dimensionality and classify sensor responses. After further optimization, only 12 sensors were required to achieve classification performance comparable to that of the larger sensor array. Although this approach is somewhat labor-intensive, it offers advantages such as portability, low cost, and suitability for field deployment.

Kangas *et al.* demonstrated the potential of array-based classification. However, the vast library of sensors complicates its application, and the reaction mechanism remains unclear. To address this issue, Zhou *et al.* revisited to the classic NBP molecule, revealing through theoretical calculations the key role of water in stabilizing reaction intermediates and elucidating the color-forming reaction pathway at the molecular level for the first time.^1^ They reported a colorimetric sensor based on 4-(*p*-nitrobenzyl)pyridine (NBP) and its derivatives, in which HD undergoes intramolecular cyclization to form an epoxysulfonium cation intermediate. This intermediate then undergoes nucleophilic substitution with the pyridine nitrogen in the NBP molecule to generate an alkylated product. Subsequently, under the action of an external alkali, the product undergoes deprotonation, triggering a significant color change from colorless/pale yellow to blue/purple. The visual detection limit for HD at room temperature is 10 ppm, which can be reduced to 2.5 ppm when heated to 60 °C. The sensor shows no significant response to nerve agents or organophosphorus pesticides. Furthermore, the authors successfully loaded NBP onto cellulose and silica gel surfaces, demonstrating its potential for solid-state detection. Compared to existing products such as M8 paper, M272 test kits, and Dräger tubes, this method offers advantages including low cost, rapid response, and ease of use, providing a highly promising colorimetric sensing strategy for the rapid on-site detection of HD.

The synthesis of NBP and its derivatives requires multi-step organic reactions. Inspired by the ability of sodium thiosulfate to capture alkyl halides, Yadav *et al.* shifted the detection system from organic probes to a more classical inorganic redox system, as presented in Fig. 5(a).^23^ They utilized HD to form an epoxysulfonium ion intermediate in water, which undergoes a nucleophilic substitution reaction with sodium thiosulfate to generate a stable Bunte salt, thereby consuming the sodium thiosulfate. In the absence of HD, iodine undergoes a redox reaction with sodium thiosulfate to form colorless iodide ions. In the presence of HD, sodium thiosulfate is consumed, allowing iodine to remain and form a deep blue complex with starch. This method has a visual detection limit of 0.02 mg for HD and exhibits excellent selectivity, showing no significant response to various interferences such as nerve agents and their simulants, acylating agents, and alkylating agents. The study further applied this method to extracts from contaminated soil, surface swab samples, and solid-phase detection using sodium thiosulfate-coated silica gel, demonstrating its potential for practical on-site detection. Compared to methods involving the synthesis of complex probes, this approach uses only three common and inexpensive reagents—sodium thiosulfate, iodine, and starch—making it simple to operate and extremely cost-effective.

**Fig. 5:**
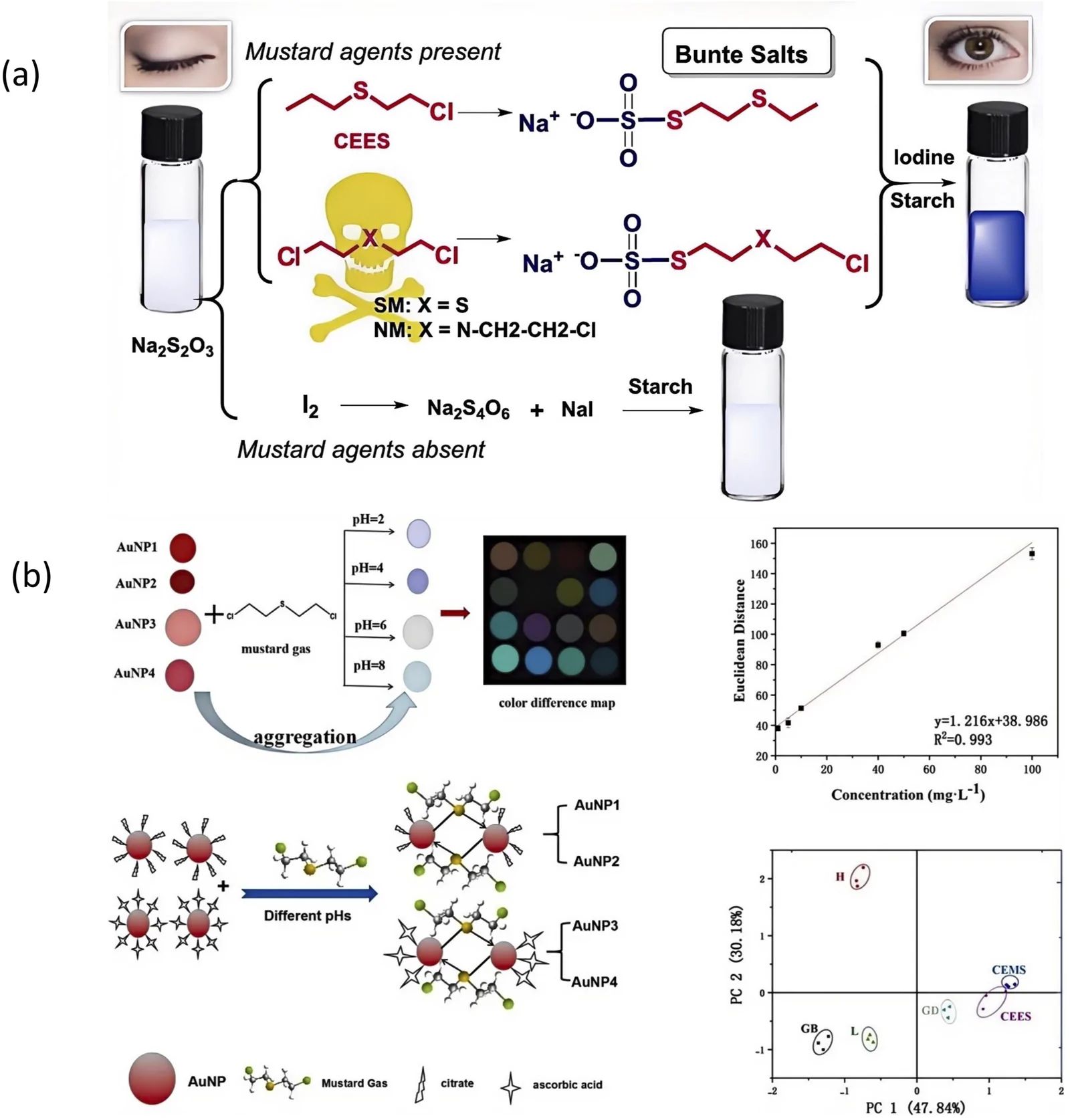
(a) The inorganic redox system designed by Yadav *et al.*; (b) the 4 × 4 colorimetric sensor array developed by Xu *et al.*

To adapt to more complex field environments, such as soil and dust, Xu *et al.* developed a 4 × 4 colorimetric sensor array based on four different particle sizes of gold nanoparticles and four pH conditions, shifting the detection platform from the molecular level to the nanomaterial level, as seen in Fig. 5(b).^5^ The sulfur atoms in the HD molecule can form Au–S bonds with the surface of gold nanoparticles, disrupting the colloidal stability of the AuNPs and inducing varying degrees of nanoparticle aggregation, resulting in visible color changes ranging from red to purple, pink or sky blue. Different pH levels affect the protonation and deprotonation state of the sulfur atom, thereby regulating the efficiency of Au–S bond. Under acidic conditions, the sulfur atom is protonated, weakening its binding ability; under alkaline conditions, it is deprotonated, enhancing its binding ability. Neutral conditions fall between these two extremes. By utilizing AuNPs of four different particle sizes, which exhibit varying degrees of response to HD, distinct color change fingerprints are generated. Extracting the RGB differences between pre- and post-reaction images and performing principal component analysis (PCA) and hierarchical clustering analysis enables effective differentiation of HD at different concentrations. This strategy offers advantages such as ease of operation, low cost, and visual feedback, providing a novel nanomaterial-based sensing approach for on-site colorimetric detection of HD.

## 6. Detection of mustard gas and its simulants using a dual-mode colorimetric and fluorescence approach

Colorimetric signals enable intuitive, naked-eye interpretation without the need for any instruments or equipment, making them ideal for rapid on-site screening in emergency situations. In contrast, fluorescent signals provide higher sensitivity and allow for quantitative analysis, making them well-suited for trace detection. The combination of these dual modes effectively addresses the limitations inherent in single-mode approaches. For example, colorimetric methods can produce false results due to ambient light interference, while fluorescence methods depend on ultraviolet light sources. By cross-validating each other, these two techniques significantly reduce the risk of false positives and false negatives, facilitating a reliable, one-step detection process that confirms results during initial screening (Table 7).

**Table 7:** Detection of mustard gas and its analogs using a dual-mode colorimetric and fluorescence method

Author	Fluorescent/chromogenic systems	Reaction type	Reaction solvent	Temperature	LOD	Response time	Fluorescent + colorimetric	Real chemical agents
Kumar *et al.*^24^	Dithiol 1 + squaraine dye (SQ)	*S*-Alkylation	Methanol	80 °C	Colorimetric: 50 µM	1 min	Blue	—
					Fluorescent: 10 µM			
Kumar *et al.*^25^	Compound 1 (acridinethione)	*S*-Alkylation	Methanol	60 °C	Colorimetric: 0.04 mg mL^−1^	1 min	Colorimetric: yellow → orange	HD
					Fluorescent: 0.005 mg mL^−1^		Fluorescent: green → yellow	
Wang *et al.*^26^	RHBT	*S*-Alkylation coupled with ESIPT	Methanol/gas phase (TLC silica gel plate)	RT/60 °C	3.19 µM (in methanol)	RT: 25 min 60 °C: 3 min/RT: 30 min (TLC silica gel plate)	Colorimetric: colorless → purple-red	—
							Fluorescent: green-yellow	
Li *et al.*^27^	SRB-NB	*S*-Alkylation	Lonic liquid ([BMIm]HSO_4_)	RT	3.0 µM	1 min	Colorimetric: colorless → red	HD
							Fluorescent: red	
Ahamed *et al.*^28^	BASO	Phosphorylation/alkylation	50% water–DMSO/gas phase (test strip)	RT	Colorimetric: 4 µM	∼1 min/1–2 min (test strip)	Colorimetric: colorless → pink	—
					Fluorescent: 0.4 µM		Fluorescent: pink	

In a 2013 study, as observed in Fig. 6(a), Kumar *et al.* reported a highly selective dual-mode colorimetric and fluorescent detection method for CEES. Under alkaline conditions, dithiols react with squarine (SQ), causing the dye to bleach.^24^ In the presence of CEES, CEES preferentially reacts with the dithiol to form a cyclic thioether compound, thereby inhibiting the reaction between the dithiol and SQ and preserving the blue color and fluorescence of SQ. This method is insensitive to nerve agent mimics and other electrophiles and has been successfully applied for detection on surfaces, in soil, and in the gas phase. Furthermore, their 2018 work overcame the drawbacks of multi-component, multi-step systems by integrating the recognition and signaling units into a single molecule.^25^ The detection mechanism involves 3,6-bis(dimethylamino)-9(10*H*)-acridinethiol (compound 1), which undergoes *S*-alkylation with the three-membered ring thiol ion formed by HD under basic conditions, subsequently restoring its conjugated structure. This produces a distinct color change from yellow to orange and a fluorescence red shift from green to yellow. The limits of detection (LODs) for visual, UV and fluorescence detection were 0.04, 0.02 and 0.005 mg mL^−1^, respectively. The method also demonstrated excellent resistance to common interferences such as nerve agents, alkylating agents, acids, and bases. Furthermore, the study successfully developed a portable TLC plate detection kit and achieved real-time detection of soil and surface contamination samples.

**Fig. 6:**
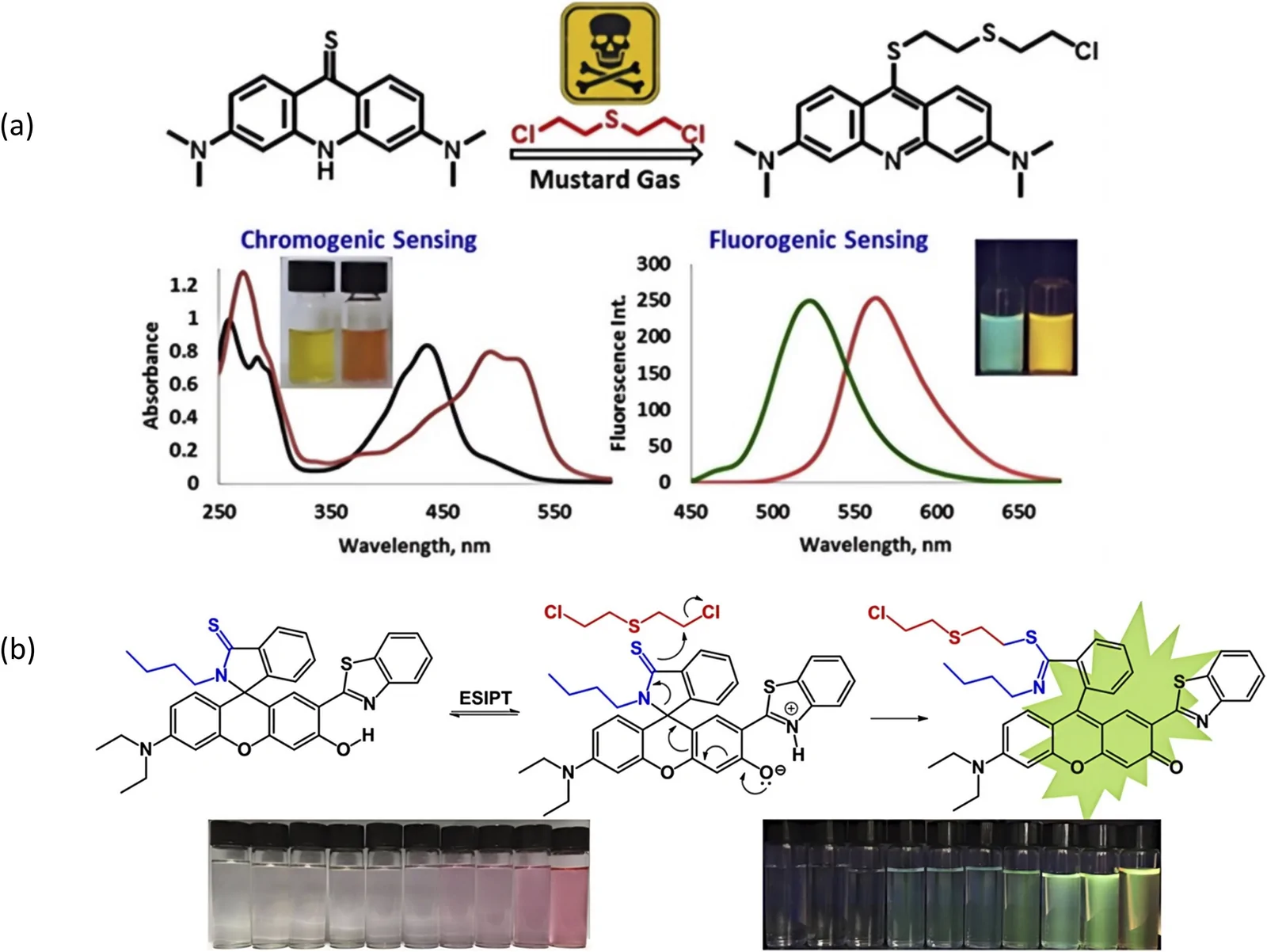
(a) The dual-mode colorimetric and fluorescent detection method reported by Kumar *et al.*; (b) RHBT reported by Wang *et al.*

However, the studies mentioned above require high temperatures for detection whereas a benzothiazole-modified rhodanol derivative (RHBT) reported by Wang *et al.* in 2018 enables CEES detection at both room temperature and elevated temperatures, as visualized in Fig. 6(b).^26^ RHBT contains a rhodanol spirothiazole thioimide structure and is colorless and non-fluorescent in its ground state. CEES undergoes an *S*-alkylation reaction with the sulfur atom in the probe, while the benzothiazole moiety and the rhodanol hydroxyl group exhibit an excited-state intramolecular proton transfer (ESIPT) effect. These mechanisms synergistically promote the opening of the spiro ring, generating a highly conjugated quinone structure that emits strong green-yellow fluorescence and produces a magenta colorimetric signal, with a detection limit of 3.19 µM. Compared to previously reported rhodamine-based probes, RHBT reduces the response time to 3 minutes at 60 °C and has been successfully applied to the detection of CEES in soil and gas phases. This work marks the first introduction of the ESIPT mechanism into rhodanone-based probes, accelerating the ring-opening reaction and providing new insights for the design of rapid-response optical sensors.

In addition to developing a detection system capable of operating at room temperature under real-world conditions, it is essential to avoid traditional organic solvents, which present challenges such as high volatility and limited solubility. Therefore, Li *et al.* pioneered the use of ionic liquids as solvents. They synthesized a fluorescent probe, SRB-NB, based on a rhodamine B-thioamide structure.^27^ SRB-NB contains a rhodamine spiro ring structure and is colorless and non-fluorescent. EES forms a selenide intermediate in the ionic liquid, which undergoes an *S*-alkylation reaction with the sulfur atom in the probe, leading to spiro ring opening and the formation of a conjugated structure, resulting in strong red fluorescence and a red colorimetric signal. After screening five imidazolium-based ionic liquids, [BMIm]HSO_4_ was found to exhibit the best sensitivity and stability for the probe, as well as the optimal optical response, with a fluorescence enhancement of up to 11.6-fold, a detection limit of 3.0 µM and a response time of approximately 1 minute at room temperature. Additionally, the study prepared a silica gel plate-loaded probe/ionic liquid system, which successfully achieved visual detection of gaseous HD and CEES under a 60 °C water bath heating condition. Color changes were observable within seconds, and the signal remained stable for over 24 hours. Mass spectrometry and DFT calculations validated the sensing mechanism of *S*-alkylation-induced ring opening. This ionic liquid-enhanced strategy provides a new approach for improving the response performance of probes in nonpolar media.

After achieving rapid response at room temperature, researchers began to pursuing lower detection limits. In 2024, Ahamed *et al.* designed and synthesized a rhodamine B- and ketone-based spiramide derivative (BASO) for the dual-mode (colorimetric and fluorescent) detection of nerve agent analogs (DCP, DCNP) and CEES.^28^ The detection principle relies on the rhodamine-lactam structure in BASO, which is colorless and non-fluorescent in its initial state. In a 50% water–DMSO mixed solvent, when the target compound acts as an electrophile and undergoes phosphorylation or alkylation reactions with the hydroxyl (–OH) and imine (–CH=N–) groups in BASO, it induces the ring-opening of the spiro-lactam, forming a conjugated, extended oxanthacene-quinone structure that generates a pink colorimetric signal and strong pink fluorescence. The same probe can simultaneously detect both nerve agent simulants and HD simulants, demonstrating multi-analyte recognition capabilities with fluorescence detection limits of 0.16 µM, 0.3 µM and 0.4 µM for DCP, DCNP, and CEES respectively. The study further demonstrated the probe's practical applications in paper-based test strips, vapor-phase detection, smartphone reading, and soil samples, and achieving five cycles of reusable performance. As the first single-molecule snail-like amide probe capable of simultaneously responding to both nerve agent and blister agent analogs, BASO provides a new approach for the rapid on-site detection of multi-target chemical warfare agents.

## 7. Quantitative comparison of sensor performance and structure–property relationship analysis

### 7.1 Quantitative comparison of key performance indicators

The core performance of optical sensors can be evaluated through four quantitative indicators: limit of detection (LOD), response time, operating temperature, and selectivity. Based on the quantitative comparison of sensors in the tables presented in the preceding sections, three regular relationships can be summarized. First, the relationship between LOD and response speed. Probes that achieve second-level responses at room temperature (such as DPXT, NDTAZ, and fluorene-based oligomers) typically exhibit LODs in the micromolar or hundred-ppb range. In contrast, probes achieving nanomolar-level LODs (such as Pr-Cou-SH and SM-Flu) require heating to 60 °C to achieve rapid responses. The root cause of this trade-off lies in the thermodynamic nature of alkylation reactions—lowering the activation energy requires heating, while enhancing intrinsic reactivity often compromises selectivity due to the reduced p*K*_a_ of the nucleophilic center. Second, the relationship between sensitivity and selectivity. Although probes based on noncovalent interactions can achieve second-level responses, their selectivity toward CEES is significantly lower than that of covalent reaction-based probes. This is because the recognition mechanism of noncovalent interactions relies on weak intermolecular forces, which are less sensitive to subtle differences in molecular structure compared to covalent bond formation. Third, the performance complementarity between colorimetric and fluorescence methods. The visual LOD of colorimetric methods is typically 2–3 orders of magnitude higher than that of fluorescence methods. However, colorimetric methods offer irreplaceable advantages in terms of reagent cost, operational simplicity, and equipment independence. The two approaches are suitable for different application levels: colorimetric methods are ideal for rapid preliminary screening, while fluorescence methods are suited for trace confirmation.

### 7.2 Systematic analysis of structure–property relationships

For thiol/thiophenolate-based probes, the p*K*_a_ value of the nucleophilic center directly determines the equilibrium concentration of the thiolate anion under neutral conditions, thereby affecting the rate of the alkylation reaction. Aliphatic thiols have p*K*_a_ values of approximately 10–11, and the proportion of thiolate anions in neutral aqueous solutions is extremely low, necessitating strongly basic conditions for effective reaction. When electron-withdrawing substituents are introduced onto the benzene ring, the p*K*_a_ of thiophenols can be reduced to 6.5–7.0, and the proportion of thiolate anions under neutral conditions can reach 5–15%, sufficient for spontaneous *S*-alkylation. The Pr-Cou-SH probe designed by Wei *et al.* (p*K*_a_ ≈ 6.8) achieves rapid reaction at room temperature without the addition of an external base, precisely exploiting this principle.^12^ For thioketone-based probes, reactivity does not directly depend on p*K*_a_ but rather on the degree of polarization of the C=S bond and the electron density on the sulfur atom—that is, introducing electron-donating groups into the heterocyclic skeleton increases the nucleophilicity of the C=S bond.

The magnitude of fluorescence signal enhancement or quenching before and after probe response depends on the degree of change in the ICT state of the fluorophore. When the nucleophilic site is directly conjugated with the fluorophore, the alkylation reaction modulates the ICT energy level by altering the electron-donating ability of the donor group. The study by Feng *et al.* systematically revealed this regularity: in the quinoline-2-thione skeleton, the introduction of diethylamino groups (strong electron donors) at the 6/7 positions endows the probe with a strong ICT character before the reaction.^11^ Following *S*-alkylation, the thioketone is converted to a thioether, the electron-donating ability is significantly enhanced, and the ICT energy gap is narrowed, resulting in a blue shift of *λ*_em_ to 520 nm and a substantial increase in fluorescence quantum yield. This “turn-on” response mechanism offers a higher signal-to-noise ratio than quenching-type probes and represents the preferred strategy for designing high-sensitivity probes.

The substituents surrounding the nucleophilic center in the probe molecule, as well as the substituents of the HD simulant itself, both influence the reaction rate. In actual agent validation, the difference in reactivity between HD and CEES partially originates from this aspect. HD bears chloroethyl groups at both termini, and its molecular conformational freedom is lower than that of CEES, resulting in a slightly higher activation energy for the formation of the sulfonium ion intermediate. At the probe molecular level, when bulky substituents are introduced at the *ortho* position of the C=S bond, the apparent activation energy for CEES to approach the reaction center increases significantly, and the room-temperature reaction rate decreases by several-fold. This factor has been less quantitatively discussed in existing studies, but its impact is more pronounced at solid-state sensing interfaces, where bulky substituents in densely packed molecular layers further hinder the diffusion and accessibility of HD molecules.

Solid-state sensing (test strips, films, *etc.*) represents the primary form of on-site detection, and the aggregated state structure of molecules directly affects sensitivity and response speed. In the dense crystalline state, the diffusion coefficient of HD molecules is four orders of magnitude lower than that in the amorphous state, meaning that the response time of crystalline probes is typically tens of minutes longer than that of amorphous probes. Therefore, in the design of solid-state probes, preference should be given to amorphous or mesoporous structures with large free volume and loose packing. One of the advantages of the MOF encapsulation strategy (such as F@Zr-BTC) is that its ordered pore channels provide rapid diffusion pathways for gas molecules.

The reaction and luminescence behavior of probes are highly dependent on the local microenvironment. Zhou *et al.* confirmed through DFT calculations that water molecules play a critical role in stabilizing the sulfonium ion intermediate in the reaction between NBP and HD.^1^ The hydrogen bonds of water can reduce the activation energy barrier of the reaction by approximately 15 kJ mol^−1^, explaining why the colorimetric reaction proceeds more rapidly under humid conditions. Similarly, Li *et al.* found that the ionic liquid [BMIm]HSO_4_, as a reaction medium, can increase the response rate of SRB-NB toward CEES by approximately 20-fold compared to conventional organic solvents, attributed to the stabilization of the alkylation reaction transition state by the high concentration of hydrogen bond donors in the ionic liquid.^27^ These results indicate that the engineering design of the reaction medium/matrix is equally important as the chemical design of the probe molecule.

### 7.3 Reflection on existing design strategies and future directions

There is an inherent contradiction between reducing the reaction activation energy to achieve rapid room-temperature responses and improving reaction specificity to achieve nanomolar-level LODs. Researchers should prioritize the indicators to be satisfied based on specific application scenarios, rather than pursuing comprehensive optimization of a single probe. The core difficulty of gas-phase detection lies in the low target concentration and rapid diffusion. The reason why porous materials such as MOFs/COFs exhibit superior gas-phase detection performance compared to molecular probes is largely attributable to the enrichment effect of their pore channels on target molecules, rather than the probe molecules themselves being more sensitive. F@Zr-BTC can effectively enrich gaseous CEES within its pores, bringing the local concentration to the solution-phase level. This strategy suggests a feasible technical pathway: integrating capture groups and signaling units within the same framework material to enable synergistic enrichment and detection, thereby achieving sensitive responses to low-concentration targets in the gas phase at room temperature.

## 8. Shortcomings and challenges in current fluorescence or colorimetric-based detection methods for mustard gas and its simulants

Currently, an increasing number of fluorescent probes and colorimetric sensors are being used to detect mustard gas and its simulant. While detection limits and response times continue to improve, many shortcomings and challenges remain.

First, the vast majority of current studies rely solely on the monochlorinated analog CEES for experiments, with very few conducting validation tests using actual mustard gas. As a monofunctional alkylating agent, CEES cannot form the three-membered ring selenide ion intermediates characteristic of mustard gas. Its reaction kinetics, electrophilic activity, and alkylation efficiency differ fundamentally from those of the actual agent. Many ultra-high-sensitivity fluorescent probes are designed and optimized for CEES. However, when applied to actual mustard gas, which exhibits lower reactivity, their response speed and sensitivity often decline significantly. The absence of a validation system using the actual chemical agent means that much of the literature data is only of comparative value and cannot be readily translated into reliable on-site detection capabilities. Second, some detection systems require heating to 60–80 °C to achieve rapid response, whereas response times at room temperature are generally too long, making them unsuitable for emergency scenarios involving real-time detection of chemical warfare agents and rapid early warning. Third, the battlefield environment requires a detection limit for mustard gas of less than 0.001 ppm (see Fig. 7 for details), whereas the detection limits of most current methods are in the micromolar range, which falls short of the requirements for rapid on-site trace detection. This fails to meet the standards for trace-level early warning. Fourth, the vast majority of studies rely on organic solvents, strong bases, or buffer systems, which increase operational complexity and hazards. Furthermore, compared to the liquid phase, the gas phase features lower target concentrations, faster diffusion rates and lacks a solvent as a mass transfer medium. Therefore, sensing materials must possess highly efficient adsorption and enrichment capabilities to capture trace molecules at sensitive sites. In the field of solid-phase detection, research on contamination models for real surfaces (concrete, wood, painted metal, fabric and rubber) remains limited and long-term storage stability testing is required. Fifth, some high-performance probes incorporate highly toxic heavy metal ions (Cd^2+^, Hg^2+^) or high-concentration ionic liquids (such as [BMIm]HSO_4_) as reaction media or signal amplification components. The use of such probes in the field poses risks of secondary contamination and operational safety hazards, failing to meet the basic requirements for on-site testing. Reagents should be low-toxicity, biodegradable, portable, and maintenance-free.

**Fig. 7:**
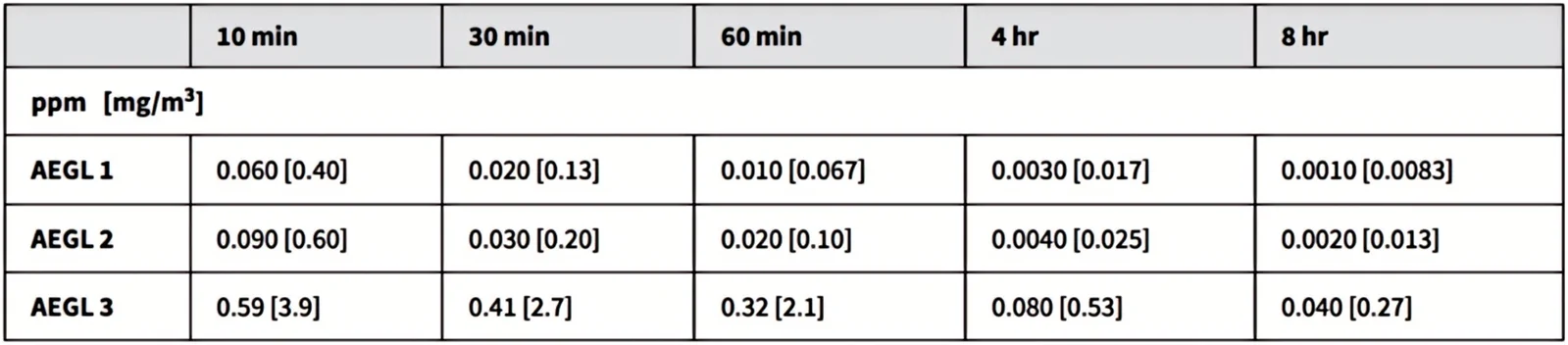
AEGL standard data sheet for mustard gas.

## 9. Key bottlenecks in the practical application of sensors

In addition to the technical challenges directly related to detection performance mentioned above, the transition of optical sensors from the laboratory to field deployment faces several key engineering and commercialization bottlenecks. First, the long-term storage stability of the sensors is the primary issue limiting their practical application. Most fluorescent probes have a short shelf life in solution, as the probe molecules may undergo photobleaching, oxidative degradation, aggregation, or aprecipitation. Although some studies have attempted to prepare solid-state films or test strips to improve stability (such as the PEO film developed by Feng *et al.*, which can be stored for several weeks at room temperature), there are very few systematic research reports on systems that can be stably stored at room temperature for at least 1–2 years.^11^ Furthermore, the reusability of sensors is a key factor affecting their cost-effectiveness. With the exception of NDTAZ, which Zheng *et al.* reported can be regenerated through water treatment, the vast majority of probes rely on irreversible alkylation reactions and become inoperable after a single use.^19^ In actual emergency scenarios, while irreversible sensors can meet the need for single-use applications, the cost advantage of reversible sensors is more pronounced in training exercises and routine monitoring. The integration with field detection devices serves as a critical bridge connecting laboratory technologies to practical operational applications. At present, various low-power, miniaturized readout devices are still in the prototype validation stage, and there is a lack of mature products that meet the protection standards for battlefield environments. The commercial economic viability cannot be overlooked. The synthesis cost of high-purity fluorescent probes is typically high, especially for those involving multi-step organic synthesis or noble metal nanomaterials, whereas military detection equipment must strictly control costs while ensuring performance.

## 10. Summary and outlook

This paper presents a systematic review of research advancements in detecting mustard gas and its simulants using fluorescent probes and colorimetric sensors. In fluorescent sensing, the technology has progressed from early multi-component, metal-dependent systems to single-component, smart molecular probes that operate without external activators. In colorimetric sensing, the focus has shifted from large-scale sensor array screening to inexpensive reagent systems with well-defined mechanisms. Bimodal systems, which integrate colorimetric and fluorescent signals, address the dual needs of rapid initial screening and sensitive confirmation.

Future breakthroughs should focus on the following areas. First, the development of rapid-response molecular probes operable at room temperature: by introducing strong electron-withdrawing groups at the *ortho* position of thiols or thiones to lower the p*K*_a_ of the sulfur atom into the neutral range, these probes can spontaneously form nucleophilic species in aqueous solutions, thereby fundamentally eliminating the need for heating or the addition of external bases. Simultaneously, we should move beyond the traditional paradigm of nucleophilic substitution reactions and explore novel recognition mechanisms and signal amplification strategies based on host–guest chemistry and catalytic reaction cycles to achieve ultrafast, ultrasensitive, and highly selective detection performance. Next, we should facilitate the transition of sensing materials from the liquid phase to solid-state film forms. By confining probe molecules and other components within solid-state matrices such as metal–organic frameworks (MOFs), covalent organic frameworks (COFs), or porous polymers, we can effectively prevent aggregation-induced fluorescence quenching and increase local molecular concentration through the pore enrichment effect, thereby significantly enhancing the sensitivity of gas-phase detection. New solid-state platforms, such as electrospun nanofiber films and flexible wearable sensor patches, also show great promise. These platforms can integrate sample enrichment, response, and signal output functions onto a single solid-state interface, enabling true “sample-and-detect” capabilities. In addition, integrating optical sensing capabilities with catalytic degradation within the same material system enables the *in situ* elimination of mustard gas while simultaneously detecting it. Researchers should, whenever, use actual HD for performance validation, systematically evaluate the performance differences between simulants and real chemical agents, and promote the establishment of unified standards for evaluating detection performance. Concurrently, efforts should be made to advance the translation of sensing materials from laboratory-scale research to practical detection equipment.

## Author contributions

Dan Meng: conceptualization, investigation, methodology, visualization, writing – original draft; Jianan Wei: data curation, formal analysis, validation, writing – review & editing; Liu Yang: conceptualization, methodology, project administration, resources, funding acquisition, validation, writing – review & editing; Shao Min Shuang: conceptualization, supervision, project administration, resources, supervision, writing – review & editing.

## Conflicts of interest

The authors declare no conflicts of interest.

## Data Availability

As this study is a review, no primary research results, software, or code are presented, and no new data were generated or analysed.
